# The Influence of Pleistocene Climatic Changes and Ocean Currents on the Phylogeography of the Southern African Barnacle, *Tetraclita serrata* (Thoracica; Cirripedia)

**DOI:** 10.1371/journal.pone.0102115

**Published:** 2014-07-23

**Authors:** Terry V. Reynolds, Conrad A. Matthee, Sophie von der Heyden

**Affiliations:** Evolutionary Genomics Group, Department of Botany and Zoology, Stellenbosch University, Matieland, South Africa; University of Illinois at Urbana-Champaign, United States of America

## Abstract

The evolutionary effects of glacial periods are poorly understood for Southern Hemisphere marine intertidal species, particularly obligatory sessile organisms. We examined this by assessing the phylogeographic patterns of the southern African volcano barnacle, *Tetraclita serrata*, a dominant species on rocky intertidal shores. Restricted gene flow in some geographical areas was hypothesized based on oceanic circulation patterns and known biogeographic regions. Barnacle population genetic structure was investigated using the mitochondrial cytochrome oxidase subunit 1 (COI) region for 410 individuals sampled from 20 localities spanning the South African coast. The mtDNA data were augmented by generating nuclear internal transcribed spacer 1 (ITS1) sequences from a subset of samples. Phylogenetic and population genetic analyses of mitochondrial DNA data reveal two distinct clades with mostly sympatric distributions, whereas nuclear analyses reveal only a single lineage. Shallow, but significant structure (0.0041–0.0065, P<0.01) was detected for the mtDNA data set, with the south-west African region identified as harbouring the highest levels of genetic diversity. Gene flow analyses on the mtDNA data show that individuals sampled in south-western localities experience gene flow primarily in the direction of the Benguela Current, while south and eastern localities experience bi-directional gene flow, suggesting an influence of both the inshore currents and the offshore Agulhas Current in the larval distribution of *T. serrata*. The mtDNA haplotype network, Bayesian Skyline Plots, mismatch distributions and time since expansion indicate that *T. serrata* population numbers were not severely affected by the Last Glacial Maximum (LGM), unlike other southern African marine species. The processes resulting in the two morphologically cryptic mtDNA lineages may be the result of a recent historical allopatric event followed by secondary contact or could reflect selective pressures due to differing environmental conditions.

## Introduction

Past climatic changes in the form of glaciations, as well as associated changes in temperature and precipitation, have been suggested as drivers of demographic change in both terrestrial and marine species. Numerous marine species have been shown to be affected by sea level fluctuations that occurred during the last 100 000 years and especially around the Last Glacial Maximum (LGM, 26 000 to 19 000 years ago) [1], [2]. Documented changes have included population bottlenecks and alterations of gene flow, as well as extinctions of local populations [3], [4], [5], [6], [7], [8]. In contrast, refugia provided a means of persistence for some populations, especially in species in the northern hemisphere [6], [9], [10], [11], [12], [13], [14]. The effects of sea level changes become less obvious in areas that had no ice cover during glaciation periods, such as southern Africa. Although the documented drop of ∼130 m in sea level caused large areas of the Agulhas Bank to emerge with the formation of the southern coastal plain [2], [15], the effects on the topology of the southern African coastline are not well known. Further, no evidence for vicariance events, such as those shown along the south-western coast of Australia as a result of the land bridge across the Bassian Isthmus, [16], [17], [18], have been documented [19]. However, a number of southern African marine species show some degree of population genetic structuring along their geographic ranges, [20], [21], [22], [23], [24], yet barriers vary between species and there are few congruent patterns [19], [25], even in closely related species [26]. The variability in these patterns makes the explanation of the processes involved in generating the population genetic structuring particularly complex [26].

Such variance in patterns also applies to the study of demographic change; some marine species have been shown to maintain demographically stable populations throughout the LGM, without significant changes in population sizes, while others show population bottlenecks with post-LGM recolonization; both scenarios seem equally plausible [8] and can be found even in closely related species [26]. To better understand the evolutionary processes that drive changes in population sizes and distributions, it is important to carefully consider the timing of population expansions and contractions [27]. Further, it has been hypothesized that species with different life-histories show different patterns in response to climatic oscillations [8], [18]. For example, sessile organisms such as suspension feeders may be more likely to persist in cooler climates due to the greater physiological costs and potential harm of mobile lifestyles under harsh conditions [8]. In addition, it has been suggested that the majority of species showing results consistent with LGM persistence are found in the lowest region of the intertidal or shallow subtidal and possess planktonic larvae [8], [18].

As with a number of studies, the link between life-history characteristics and population genetic structure is tenuous at best. Evidence from direct and indirect estimates of dispersal suggest that larvae consistently disperse on smaller spatial scales than expected based on their pelagic larval duration (PLD) [28], [29], [30]. Species with planktonic larval stages do not always result in panmictic populations [21], [31], [32], [33], [34], and species with poor dispersal abilities, such as direct developers or species that occupy small home ranges can show connectivity [16], [35]. Furthermore, a long PLD can contribute towards genetic panmixia [35], [36], [37], [38], [39], [40], [41], but known barriers to gene flow, which may be linked to oceanic circulation in a region and or to historical processes [3], [22], [33], [36], [42], [43], [44], can also play significant roles in shaping genetic patterns.

**Figure 1:**
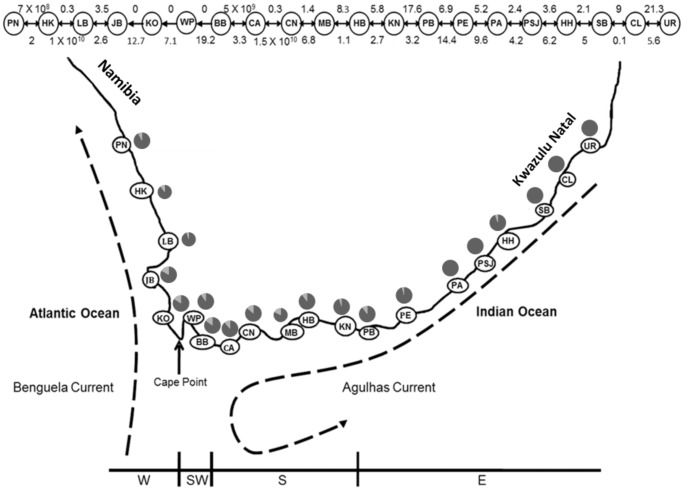
Map showing the distribution of *Tetraclita serrata* from KwaZulu-Natal to Namibia. Sampled localities (see Table 1) for *Tetraclita serrata* are shown in addition to coastal regions [west (W), southwest (SW), south (S), east (E)] in which samples were collected. These coastal regions correspond broadly with the Namaqua, south western Cape, Agulhas and subtropical Natal Bioregions, respectively [47]. The frequency of Clade A and Clade B within each sampling locality are indicated using pie charts. The major ocean currents are shown. The direction and intensity of gene flow between sampled localities, and the overall direction of gene flow are indicated by arrows; mutation-scaled migration rates between localities are indicated above these arrows.

In an attempt to contribute to the growing body of literature describing genetic patterns in southern African marine species and to determine the effect of the LGM on an intertidal organism, we chose the volcano barnacle, *Tetraclita serrata*, one of the dominant invertebrates of the mid intertidal zone with a wide distribution ranging from KwaZulu-Natal (east coast of South Africa), extending into Namibia (west coast of southern Africa; Fig. 1). Although this range spans at least four of the five biogeographic provinces described for the region, the species is more commonly found on the south and east coasts of South Africa (Fig. 1) [45]. A systematic study, incorporating both molecular and morphological data, suggests that *T. serrata* consists of two sympatrically distributed populations in South Africa [46]. Some conclusions were made on the demography of the species based on molecular diversity indices, but in the absence of molecular dating, the effect of the LGM could not be established. Furthermore, although hypotheses on gene flow were presented, no gene flow analyses were conducted.

The present study provides a more in-depth phylogeographic consideration, based on a considerably larger data set, to elucidate the intraspecific variation of *T. serrata* across sampling localities along the South African coastline. As barnacles are obligate sessile organisms with planktotrophic larvae, it is hypothesized that the longevity and dispersal capabilities of juvenile stages and the influence of the associated current systems may dictate the dispersal and genetic connectivity of the species. Given their position in the mid-intertidal, it is also postulated that *T. serrata* was not severely affected by the LGM (see [8], [18]). To test these hypotheses, the cytochrome oxidase subunit 1 (mtDNA COI) and the nuclear internal transcribed spacer 1 (nDNA ITS1) were used to document the phylogeographic structure of *T. serrata*; gene flow patterns and demographic changes of the species were investigated using the mtDNA data.

## Materials and Methods

### Study area

The oceanographic patterns of southern Africa are complex and dynamic with seasonal effects on offshore, as well as coastal marine species [47]. The area is governed by two major boundary currents; the cold-water Benguela Current, which flows northwards along the west coast of the continent, and the warm-water Agulhas Current, which moves southwards along the east coast of South Africa (Fig. 1). The diverse oceanographic and biological regimes along the South African coastline have been recognized to fall into five distinct biogeographic provinces, each characterized by a unique suite of potentially physiologically-adapted plant and animal assemblages [48].

### Collection of material

A total of 410 adult individuals of *T. serrata* were randomly collected across 20 intertidal sites (Fig. 1; Table 1). Sampling localities span most of the distribution and have been partitioned according to coastal region (west, south-west, south and east; Table 1), which broadly correspond to four of the five coastal bioregions as defined by Griffiths *et al*. [48]. All samples were collected in the form of whole individuals and stored in 100% ethanol until DNA extraction. Sampling was carried out under permits from the Department of Agriculture, Forestry and Fisheries (RES2010/01, RES2011/12).

**Table 1:** Abbreviation codes for localities, geographic coordinates (latitude & longitude), number of individuals (N), number of haplotypes (Nh), number of private haplotypes (Nph), number of polymorphic sites (Ps), mismatch distribution parameters [Sum of Squared Deviations statistic (SSD), Harpending's raggedness indices (H'sR_i_)], neutrality test values (Fu's F_s_), and probabilities (*P*) for neutrality test values for analyzed populations of *T. serrata* using the COI gene.

Population	Code	Lat/Long	N	Nh	Nph	Ps	SSD	H'sR_i_	Fu's F_s_	*P*
**West Coast**										
Port Nolloth	PN	29.15–16.51	13	10	5 (50%)	46	0.0157	0.0291	–0.8793	0.306
Hondeklip	HK	30.19–17.16	19	16	8 (50%)	54	0.0167	0.0292	–4.0279	0.044
Lamberts Bay	LB	32.06–18.17	20	17	8 (47%)	49	0.0098	0.0427	–9.2934	0
Jacob's Bay	JB	32.57–17.52	24	19	9 (79%)	52	0.0256	0.0313	–4.8408	0.045
Kommetjie	KO	34.08–18.19	19	15	11 (73%)	52	0.2563	0.0143	–1.7746	0.192
**South-West Coast**										
Wooley's Pool	WP	34.13–18.45	21	17	10 (59%)	61	0.0125	0.0204	–3.6269	0.061
Betty's Bay	BB	34.21–18.55	20	19	14 (74%)	54	0.0192	0.0167	–6.8185	0.007
**South Coast**										
Cape Agulhas	CA	34.85–20.75	18	16	9 (56%)	57	0.0138	0.0307	–4.4470	0.036
Cape nfanta	CN	34.27–20.51	22	20	10 (50%)	50	0.0181	0.0174	–7.5427	0.006
Mossel Bay	MB	34.11–22.09	23	21	10 (48%)	53	0.0229	0.0253	–7.5982	0.008
Herold's Bay	HB	34.03–22.23	20	16	5 (31%)	52	0.0115	0.0290	–3.9719	0.066
Knysna	KN	34.04–23.01	20	16	9 (56%)	48	0.0567	0.0185	–6.1829	0.01
Plettenberg Bay	PB	34.03–23.22	24	19	11 (58%)	53	0.0119	0.0255	–5.8744	0.014
**East Coast**										
Port Elizabeth	PE	33.58–25.40	24	20	8 (40%)	54	0.0061	0.0230	–8.0102	0.001
Port Alfred	PA	33.36–26.54	19	13	4 (31%)	20	0.0080	0.0421	–6.5499	0.003
Port St John's	PST	31.38–29.31	20	18	8 (44%)	27	0.0036	0.0218	–14.0482	0
Haga Haga	HH	32.45–28.14	19	16	7 (44%)	52	0.0052	0.0179	–6.3005	0.006
Shelly Beach	SB	30.48–30.24	21	18	10 (56%)	30	0.0023	0.0186	–13.9663	0
Clansthal	CL	30.14–30.47	20	18	9 (50%)	25	0.0088	0.0513	–15.0865	0
Unhlanga Rocks	UR	29.42–31.04	24	17	5 (29%)	25	0.0018	0.0177	–9.8098	0
**Total**			410	213			0.004	0.009		

### DNA extraction, PCR amplification and sequencing

Genomic DNA was extracted from cirral tissue using the Wizard SV genomic DNA purification system (Promega). PCR amplification and sequencing of a fragment of the COI gene was initially carried out using universal primers [49]. The data generated by these primers were used to design species-specific degenerate primers which were subsequently used in all reactions (COI-TsF: 5′-CGG RGC TTG ATC CGC YAT RGT MGG-3′ and COI-TsR: 5′-GCT CCG GCT AAA ACT GGA AKH G-3′). Nuclear DNA data were generated from a subset of samples by amplifying the ITS1 region (including partial sequences of the 18S and 5.8S genes that flank the ITS1 region) using the primers of Bass *et al*. [50]. Amplification was performed in 50-µl reactions containing 4 µl of 10–100 ng DNA, 2.5 µl each primer (0.1 mM), 5 µl 10xPCR reaction buffer (Super-Therm), 5 µl dNTPs (0.2 mM, Thermo Scientific), 4 µl Mg^2+^ (Super-Therm), 0.2 µl Taq polymerase (Super-Therm) and 13.4 µl distilled water. PCR reactions were carried out in an ABI GeneAmp 2700 automated thermocycler with the following conditions: an initial denaturation step at 94°C for 3 min, followed by 35 cycles of denaturing at 94°C for 30 s, annealing (for 30 s) and an extension at 72°C for 45 s, ending with a final extension at 72°C for 5 min. PCR annealing temperatures were set at 58°C and 56°C for the COI and ITS1 loci, respectively. The amplified products were gel separated and purified using the Wizard SV Gel PCR Clean-Up System (Promega). Purified products were cycle-sequenced using BigDye (Applied Biosystems [ABI]) and analyzed on a 3730 automated sequencer (ABI).

### Genetic diversity

Sequences were manually inspected and edited using BioEdit v7.0.9.1 [51], and the mtDNA nucleotides were translated into protein codons to confirm functionality. All haplotypes have been deposited in GenBank with the following accession numbers KC935448-KC935857 (COI) and KC967663-KC967682 (ITS1).

Haplotype designation of the nuclear DNA data was performed in PHASE, using a coalescent-based Bayesian method [52] as implemented in DnaSP v5.1 [53]. A total of 1 000 000 iterations were performed using default settings. Arlequin 3.5 [54] was used to calculate the number of polymorphic sites and the haplotype (*h*) and nucleotide (*π*) diversities for both marker types.

### Analyses of population structure

Population genetic structure was investigated for the COI data using analyses of molecular variance (AMOVA) in Arlequin 3.5 [54]. Differentiation across sampling localities was examined through pairwise Φ_ST_ comparisons. Bayesian geographical structuring was investigated using BAPS v5.4 [55], where the geographic coordinates of each sampling locality were obtained from Google Earth. To investigate geographic distance as a contributor towards genetic structure, isolation by distance [56] was tested using a Mantel test [57] as implemented in Arlequin 3.5 with 1000 permutations. The geographical distances between sampling localities were measured as the shortest land-sea interface in Google Earth.

Statistical parsimony haplotype networks were constructed using TCS 1.21 [58] and haplotypes were connected using a 95% confidence interval. The higher level clustering of clades that were not connected in the haplotype networks was depicted using Bayesian analyses, coupled with the Markov Chain Monte Carlo (BMCMC) inference, conducted in MrBayes v3.04b [59]. The most likely (best probabilistic) model of sequence was determined using the Bayesian Information Criteria (BIC) and Akaike Information Criteria (AIC) for the COI and ITS1 gene, respectively, as employed in the program jModelTest v0.1.1 [60]. Bayesian analyses of the COI and ITS1 sequence data were run for 10 million and five million generations, respectively, with a burn-in of 25%. For each gene, two different runs were conducted to test for convergence, each with four chains and sampling frequency of 1000. Thereafter, the post-burn in samples of the two runs were combined and a 50% majority-rule consensus tree was constructed in MrBayes v3.04b. Clade support was assessed using posterior probabilities for the Bayesian tree and only values ≥0.95 were regarded as robust support. *Tetraclita squamosa* and *Tetraclita pacifica* were used as outgroup taxa (GenBank accession numbers DQ647770 and DQ363746, and DQ363680 and DQ363740, respectively).

### Demographic history

Arlequin 3.5 was used to test the influence of historical sea level changes on the demography of *T. serrata*. Fu's F_s_ test [61] was performed to test for deviations from neutrality. To confirm that departures from neutrality were caused by demography and not by natural selection, the McDonald-Kreitman (MK) test was performed in DnaSP v5.1, using *T. squamosa* and *T. pacifica* (GenBank accession numbers DQ363697–DQ363706 and DQ363686–DQ363695) as outgroups, respectively. This was followed by a mismatch distribution to test for population expansion and to estimate parameters needed to date possible population expansions [54], [62]. The formula T = τ/2 µ*k* [62] was used for dating approximations. As the exact mutation rates and generation times for *T. serrata* are unknown, we used the generation times of various barnacle species, ranging from one to two years [8], [32], [63], [64], and two published COI lineage mutation rates for barnacles (1.55×10^−8^ [65] and 2.76×10^−8^ substitutions per site per generation [18]).

A Bayesian Skyline Plot (BSP) [66] was constructed using BEAST v1.8 [67] to estimate changes in population size over time and to date a possible population expansion. The unique haplotypes of each COI data set (Clades A and B) were run for 300 million and 200 million generations, respectively, under a GTR+I+G nucleotide substitution model with individual parameters estimated from the data and a constant skyline model with five groups. For each BSP, prior distributions for the root height of the population were notified by initial estimates from the mutation rates used in the mismatch distribution analyses. The chain was sampled every 100 000 generations and the first 10% was discarded as burn-in. Trace plots were inspected to assess convergence, mixing and stationarity of the MCMC process in Tracer v1.6 [68]. The effective sample sizes were checked and confirmed to be ≥200 in order to avoid autocorrelation of parameter sampling.

### Connectivity and direction of relative gene flow between sampling localities

The program Migrate 3.1.6 [69] was used to calculate the relative migration rates between sampling localities inferred from the mtDNA data. Given the linear South African coastline, a stepping-stone model was used [70]. The settings were as follows: 10 short-chains, each with a total of 2,500 generations and a sampling increment of 20 generations, and 3 long-chains, each with a total of 50,000 generations and a sampling increment of 50 generations. The first 10,000 genealogies were discarded (burn-in). An adaptive heating scheme with four chains (starting values of 1.00, 1.50, 3.00 and 6.00) and a swapping interval of one was used to ensure that efficient mixing occurred. For the other settings, default values were implemented. The analyses were run twice to ensure congruence.

## Results

### Genetic diversity and population structure

#### Mitochondrial DNA analyses

**Figure 2:**
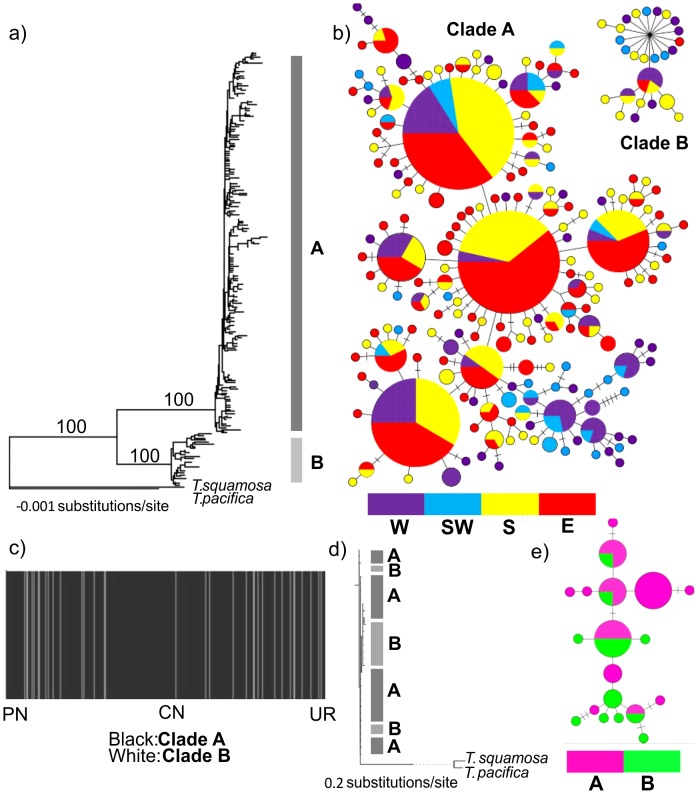
MtCOI data as visualized in (a) a Bayesian tree (constructed in MrBayes MrBayes v3.04b), (b) haplotype network (constructed in TCS 1.21), and (c) spatial clustering of individuals (constructed in BAPS v5.4). NuITS1 data is visualized in (d) a Bayesian tree and (e) a haplotype network. In b & e, the size and shading of the circles corresponds to the frequency of that haplotype and the coast from which the individual was sampled (west  = W, south-west  =  SW, south  = S, east  = E). Each line represents one mutational step and perpendicular lines represent additional mutational steps. Each sampled haplotype is colour-coded according to the coast that the individual was sampled in. In c), each vertical bar represents one individual grouped by region and location in the same order as Table 1. The shading of the vertical bars indicates to which group they are assigned by the BAPS analysis.

The COI data analyses were based on a final alignment of 499 bp for 410 individuals. The maximum parsimony network, Bayesian tree and spatial clustering of all individuals (BAPS) indicate that *T. serrata* comprises two genetically distinct groups with strong nodal support. The two genetic assemblages occur sympatrically (Fig. 2a–c). These haplogroups represent two reciprocally monophyletic lineages, both showing Bayesian Posterior Probabilities of 1.00 (BPP). These are labeled Clades A and B (Fig. 2) and are separated by a mtDNA sequence divergence of ∼8%. In proportion to Clade A, the frequency of individuals belonging to Clade B is markedly smaller (a sampling ratio of 12∶1, Fig.1).

**Figure 3:**
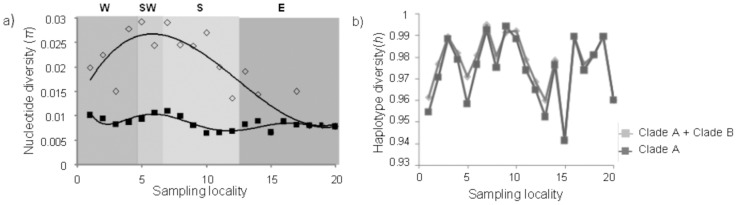
Molecular diversity indices across the distribution range of *T. serrata* include (a) nucleotide diversity and (b) haplotype diversity values for the entire data set (Clade A+ Clade B, represented by diamonds) and for Clade A individuals (represented by squares).

Within Clade A, haplotype diversity was 0.979 (±0.003), with 187 haplotypes detected, and within Clade B it was 0.976 (±0.019), with 26 haplotypes detected. The nucleotide diversity of the complete data set was 0.019 (±0.01); generally mtDNA genetic diversity was highest on the south-west and south coasts, with a progressive decrease towards the east coast and the lowest values on the west coast (Fig. 3a). With the removal of Clade B, the nucleotide diversity values are lower, with the south-west coastal region generally being the highest (Fig. 3). Although the haplotype diversities determined for each locality for the entire data set (Clade A and Clade B individuals) were generally high (>0.94, Fig. 3b), on a broader scale, sampled localities on the south-west coast, as well as the closest localities sampled on either side of the south-west coast region, namely Jacob's Bay, Kommetjie and Cape Agulhas, each had more than 50% unique haplotypes (Table 1). In contrast, those haplotypes shared among regions occur more frequently along the east coast than in the other regions (i.e. fewer private haplotypes, less than 45%).

Within Clade A, the haplotype network (Fig. 2b) shows that a considerable amount of haplotype sharing is found along the east coast. Also, west and south-west coast sampled localities group together and are more divergent, suggesting spatial genetic structure. In Clade B, the majority of haplotypes are representative of individuals sampled along the west, south-west and south coasts, with only two Clade B individuals sampled as far as Haga Haga. Many of the haplotypes within this haplogroup are separated from the common haplotype by an intermediate “unsampled or extinct” haplotype (Fig. 2b).

AMOVA suggests shallow, but significant, genetic differentiation among the 20 sampled localities (Φ_ST_ = 0.035, P<0.01); most of the variation was present within sampled localities (96.44% for the entire data set). When sampling localities were partitioned into their respective coastal regions (Table 1), AMOVA revealed significant differentiation among groups (Φ_CT_ = 0.041, P<0.01). When the individuals belonging to Clade B (Fig. 2b) were removed from subsequent analyses, the Φ_ST_ value increased (0.065, P<0.01). For the entire data set, pairwise Φ_ST_ analysis showed that Kommetjie was significantly differentiated from all localities east of Cape Agulhas (south coast), with Φ_ST_ values ranging from 0.09 to 0.23 (south coast, Table 2). Kommetjie was also found to be significantly differentiated from most of the localities sampled on the west coast (Table 2). Betty's Bay was found to be significantly differentiated from all localities east of Herold's Bay (south coast), with Φ_ST_ values ranging from 0.1 to 0.19 (Table 2). Within Clade A, Kommetjie and Betty's Bay were significantly differentiated from all sampling localities, except from each other and from Port Nolloth, with Φ_ST_ values ranging from 0.07 to 0.34 (Table 2). Due to the relatively small sample size represented by individuals of Clade B, pairwise Φ_ST_ analysis was not conducted for this clade.

**Table 2:** Pairwise ΦST values among sampling localities for *T. serrata* using the COI gene.

Location	PN	HK	LB	JB	KO	WP	BB	CA	CN	MB	HB	KN	PB	PE	PA	PST	HH	SB	CL	UR
PN	―	**0.17**	**0.17**	**0.22**	0.01 (0.35)	**0.09**	0.00 (0.32)	**0.13**	**0.22**	**0.28**	**0.24**	**0.25**	**0.26**	**0.22**	**0.25**	**0.21**	**0.22**	**0.25**	**0.23**	**0.21**
HK	0.04 (0.14)	―	0.02 (0.90)	0.01 (0.66)	**0.25**	0.01 (0.32)	**0.14**	0.02 (0.75)	0.03 (0.92)	0.00 (0.41)	0.01 (0.58)	0.00 (0.41)	0.01 (0.41)	0.01 (0.78)	0.00 (0.33)	0.03 (0.94)	0.01 (0.65)	0.00 (0.38)	0.00 (0.37)	0.02 (0.79)
LB	0.06 (0.09)	0.02 (0.65)	―	0.01 (0.06)	**0.24**	0.01 (0.68)	**0.13**	0.02 (0.85)	0.00 (0.51)	0.00 (0.41)	0.00 (0.40)	0.00 (0.42)	0.00 (0.44)	0.02 (0.78)	0.00 (0.47)	0.01 (0.78)	0.01 (0.60)	0.00 (0.39)	0.01 (0.66)	0.01 (0.32)
JB	0.06 (0.12)	0.03 (0.72)	0.02 (0.24)	―	**0.29**	0.02 (0.16)	**0.20**	0.01 (0.64)	0.01 (0.70)	0.02 (0.17)	0.01 (0.42)	0.02 (0.79)	0.01 (0.69)	0.02 (0.79)	0.01 (0.40)	0.01 (0.63)	0.01 (0.69)	0.00 (0.44)	0.02 (0.91)	0.01 (0.53)
KO	0.02 (0.42)	**0.08**	**0.11**	**0.08**	―	**0.13**	0.02 (0.14)	**0.23**	**0.31**	**0.34**	**0.34**	**0.33**	**0.30**	**0.29**	**0.34**	**0.29**	**0.31**	**0.31**	**0.31**	**0.32**
WP	0.01 (0.54)	0.02 (0.60)	0.11 (0.48)	0.00 (0.34)	0.02 (0.30)	―	**0.07**	0.01 (0.54)	0.02 (0.12)	0.03 (0.08)	0.04 (0.12)	0.03 (0.11)	0.03 (0.11)	0.02 (0.11)	**0.04**	0.02 (0.09)	0.02 (0.14)	0.02 (0.14)	0.02 (0.15)	0.02 (0.05)
BB	0.02 (0.54)	**0.05**	0.08 (0.06)	0.06 (0.06)	0.03 (0.80)	0.01 (0.36)	―	**0.13**	**0.20**	**0.22**	**0.23**	**0.22**	**0.21**	**0.20**	**0.22**	**0.19**	**0.19**	**0.22**	**0.20**	**0.21**
CA	0.01 (0.30)	0.04 (0.96)	0.02 (0.63)	0.03 (0.67)	0.05 (0.08)	0.03 (0.79)	0.03 (0.14)	―	0.01 (0.69)	0.03 (0.06)	0.00 (0.51)	0.01 (0.24)	0.03 (0.08)	0.00 (0.58)	0.02 (0.18)	0.01 (0.76)	0.02 (0.82)	0.01 (0.32)	0.01 (0.77)	0.02 (0.90)
CN	0.06 (0.14)	0.04 (0.97)	0.00 (0.32)	0.03 (0.90)	**0.10**	0.01 (0.35)	**0.07**	0.03 (0.88)	―	0.01 (0.39)	0.01 (0.61)	0.01 (0.52)	0.01 (0.27)	0.01 (0.67)	0.01 (0.25)	0.02 (0.87)	0.02 (0.84)	0.00 (0.37)	0.00 (0.50)	0.02 (0.94)
MB	0.08 (0.12)	0.02 (0.56)	0.02 (0.24)	0.03 (0.73)	**0.09**	0.01 (0.19)	0.07 (0.08)	0.02 (0.34)	0.03 (0.84)	―	0.01 (0.25)	0.01 (0.73)	0.01 (0.57)	0.02 (0.93)	0.02 (0.79)	0.02 (0.9)	0.02 (0.75)	0.02 (0.87)	0.00 (0.44)	0.02 (0.12)
HB	0.07 (0.08)	0.04 (0.84)	0.02 (0.51)	0.02 (0.52)	**0.12**	0.01 (0.54)	0.09 (0.05)	0.03 (0.57)	0.03 (0.69)	0.01 (0.46)	―	0.02 (0.88)	0.02 (0.17)	0.01 (0.67)	0.02 (0.78)	0.02 (0.74)	0.02 (0.83)	0.01 (0.78)	0.01 (0.67)	0.01 (0.50)
KN	**0.10**	0.02 (0.50)	0.03 (0.71)	0.01 (0.25)	**0.15**	0.01 (0.30)	**0.12**	0.01 (0.38)	0.00 (0.44)	0.02 (0.23)	0.03 (0.89)	―	0.01 (0.69)	0.02 (0.91)	0.02 (0.89)	0.01 (0.63)	0.02 (0.94)	0.02 (0.88)	0.01 (0.75)	0.00 (0.38)
PB	0.09 (0.08)	0.03 (0.78)	0.02 (0.75)	0.01 (0.35)	**0.12**	0.00 (0.38)	**0.10**	0.01 (0.46)	0.01 (0.39)	0.00 (0.23)	0.02 (0.69)	0.02 (0.82)	―	0.01 (0.57)	0.01 (0.23)	0.00 (0.33)	0.00 (0.47)	0.00 (0.58)	0.00 (0.50)	0.02 (0.13)
PE	**0.11**	0.01 (0.54)	0.03 (0.94)	0.02 (0.32)	**0.16**	0.01 (0.34)	**0.13**	0.01 (0.36)	0.00 (0.32)	0.02 (0.14)	0.02 (0.69)	0.03 (0.96)	0.02 (0.86)	―	0.02 (0.97)	0.02 (0.97)	0.02 (0.98)	0.02 (0.99)	0.03 (0.99)	0.00 (0.34)
PA	**0.17**	0.03 (0.10)	0.01 (0.62)	**0.07**	**0.22**	**0.06**	**0.18**	**0.04**	0.06 (0.07)	0.08 (0.05)	0.01 (0.42)	0.01 (0.89)	0.02 (0.19)	0.02 (0.92)	―	0.02 (0.74)	0.02 (0.78)	0.01 (0.82)	0.01 (0.78)	0.01 (0.23)
PST	**0.15**	0.01 (0.43)	0.02 (0.91)	0.06 (0.10)	**0.20**	**0.04**	**0.17**	0.02 ((0.22)	0.04 (0.26)	0.06 (0.07)	0.01 (0.51)	0.01 (0.90)	0.01 (0.47)	0.02 (0.96)	0.02 (0.8)	―	0.03 (0.96)	0.02 (0.94)	0.02 (0.84)	0.01 (0.75)
HH	**0.09**	0.02 (0.72)	0.03 (0.85)	0.01 (0.28)	**0.14**	0.00 (0.31)	**0.11**	0.02 (0.57)	0.01 (0.52)	0.01 (0.37)	0.03 (0.97)	0.04 (0.96)	0.02 (0.72)	0.03 (0.98)	0.01 (0.81)	0.02 (0.96)	―	0.02 (0.85)	0.02 (0.90)	0.02 (0.86)
SB	**0.18**	0.03 (0.08)	0.00 (0.42)	0.07 (0.10)	**0.22**	**0.05**	**0.18**	0.04 (0.07)	0.06 (0.11)	0.08 (0.06)	0.02 (0.26)	0.01 (0.86)	0.01 (0.32)	0.01 (0.87)	0.01 (0.71)	0.02 (0.93)	0.01 (0.68)	―	0.02 (0.84)	0.01 (0.10)
CL	**0.16**	0.03 (0.19)	0.01 (0.84)	0.06 (0.05)	**0.21**	**0.04**	**0.17**	0.03 (0.11)	0.05 (0.14)	0.08 (0.09)	0.02 (0.49)	0.01 (0.78)	0.01 (0.27)	0.02 (0.92)	0.01 (0.81)	0.02 (0.79)	0.01 (0.83)	0.02 (0.86)	―	0.00 (0.39)
UR	**0.15**	0.03 (0.18)	0.00 (0.24)	0.07 (0.05)	**0.23**	**0.06**	**0.19**	0.03 (0.10)	0.05 (0.19)	**0.09**	0.02 (0.14)	0.00 (0.37)	0.03 (0.07)	0.00 (0.32)	0.01 (0.23)	0.02 (0.72)	0.01 (0.69)	0.01 (0.19)	0.00 (0.28)	―

Clade A and the entire data set phi-ST values are indicated above and below the diagonal, respectively. P-values are shown in brackets and values in bold are significant (P<0.05).

#### Nuclear DNA analyses

The aligned sequence data for ITS1 were 426 bp in length, with 31 unique alleles and four indels detected. The ITS1 data displayed a high haplotypic diversity of 0.960 (±0.019) and a low nucleotide diversity of 0.010 (±0.005), which is comparable with that of the mtDNA data. No significant structure was obtained and Clades A and B formed by the mitochondrial DNA analyses collapsed into a single haplogroup (Fig 2d, e).

### Isolation by distance

A significant correlation between geographic distances and genetic differentiation was obtained for the entire *T. serrata* data set (Mantel test, Z = 8.925, *P*<0.01), revealing a larger Z-value of 14.176 (*P*>0.05), which was not significant, with the removal of Clade B. The Mantel test was found to be non-significant when isolation by distance (IBD) was tested within each coastal region [west coast, Z = 0.119 (*P*>0.05); south coast, Z = −0.035 (*P*>0.05); east coast, Z = −0.096 (*P*>0.05)]. South-west coast localities were not included in the test because only two localities had been sampled in that region.

### Migration estimates between sampling localities

Within the sampling range of *T. serrata*, the coalescent stepping-stone model revealed migration rates that were largely bi-directional in the south and east coastal regions, whereas a more asymmetrical gene flow pattern was evident in a westerly direction between Mossel Bay and Hondeklip Bay (with the exception between Betty's Bay and Cape Agulhas) (Fig. 1). Interestingly, no southward gene flow was detected between Jacob's Bay and Betty's Bay, around Cape Point (Fig. 1).

### Demographic history

For the mtDNA data, Fu's F_s_ revealed negative values for the entire data set (although it was not significant for all sampling localities; Table 1), Clade A (F_s_ = −17.858, P<0.01) and Clade B (F_s_ = −25.065, P<0.01), respectively. The MK test was not significant for *T. serrata* when *T. squamosa* (G-value  = 0.841, P>0.05) was used as an outgroup. The more closely-related species *T. pacifica* could not be used for this test because of the lack of nonsynonymous fixed differences with *T. serrata* in the sequenced gene region. A pattern of population expansion was confirmed for both clades by the observed unimodal mismatch distributions (not shown), which were not significantly different from the Poisson distributions predicted by a sudden expansion model. This hypothesis of population expansion is supported by both the Sum of Squared Deviations (*P*>0.05) and Harpending's raggedness indices (*P*>0.05, Table 1), which tests for a fit to a unimodal mismatch distribution.

**Figure 4:**
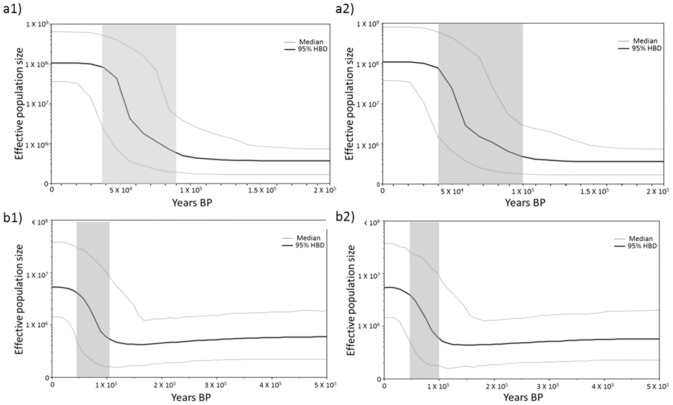
Bayesian Skyline Plot of effective population size through time based on the mtDNA sequence data for Clade A (a1 and a2) and Clade B (b1 and b2), using the mutation rates 3.1/My (equivalent to 1.55×10^−8^ substitutions per site per generation; a1 and b1) and 5.52/My (a2 and b2), respectively. The shaded area indicates the period of population expansion.

The time since population expansion was calculated for mtDNA Clades A and B (Table 3). Analyses of Clade A suggest the time since expansion between ∼40 000 and 145 000 years ago. Similarly, for Clade B, the time since expansion was calculated to be between ∼46 000 and 164 000 years ago. Bayesian Skyline Plot analyses reveal dates similar to the mismatch distribution analyses. Analyses of Clade A show a population expansion took place between ∼40 000 and 100 000 years ago, while analyses of Clade B show a population expansion took place between ∼50 000 and 100 000 years ago (Fig. 4).

**Table 3:** The time since expansion for the entire mtDNA data set and mtDNA Clades A and B using expansion times (τ) calculated in Arlequin 3.1 and published COI lineage mutation rates.

	Mutation rate	Time since expansion	
		k = 1	k = 2
**Entire data set**			
τ = 3.457	1.55×10^−8^	111,740	55,870
	2.76×10^−8^	62,753	31,376
**Clade A**			
τ = 4.498	1.55×10^−8^	145,388	72,694
	2.76×10^−8^	81,650	40,825
**Clade B**			
τ = 5.078	1.55×10^−8^	164,135	82,067
	2.76×10^−8^	92,178	46,089

Time since expansion and generation time (k) is represented in years.

## Discussion

### Within-population diversity

Genetic diversity indices varied across the sampling range, with striking differences between sampling areas; generally, diversities were highest on the west and south-west coasts, and they decreased towards the eastern localities. A distinct peak in diversity was found for the south-west coast (Fig. 3). There were also notable differences in the distribution of private/unique haplotypes, with a higher number of private haplotypes found in the south-west coastal region (Table 1).

Regions of higher genetic diversity can be explained by several scenarios. Genetic variation can accumulate over evolutionary time if a region contains an area of high local retention or larval accumulation, especially where larvae accumulate in the near-shore environment [29], [71], [72]. High genetic diversity is also expected in a region where a large population has been maintained over evolutionary time, possibly due to the stability of the habitat [73]. If contemporary population size is an indicator of evolutionary history, then this will have contributed to the high genetic diversity as *T. serrata* usually maintains large population sizes on most rocky shores [45], [74], [75]. Irrespective of population size, regions of higher genetic diversity can also signal refugial areas during glacial low sea-level stands [76]. It can also be a signal of the absence of strong directional or stabilizing selection, as both mechanisms have been shown to reduce genetic diversity [77]. Populations at or near refugia should retain genetic diversity and may also be indicated by the presence of ancestral haplotypes [78]. Interestingly, Betty's Bay on south-west coast, possesses the highest combination of haplotype and nucleotide diversity (Tables 1, 2). A study on the commercially exploited rock lobster, *Jasus lalandii*, also identified the region between Betty's Bay and Kommetjie as one of high diversity [21], but the processes driving this area to be more genetically diverse than *others are not understood.*

### Genetic structure of *Tetraclita serrata*

Population genetic structure of *T. serrata* is characterized by a significant AMOVA and a strong signal of isolation by distance for the mtDNA data. An important result was that *T. serrata* is comprised of two genetically distinct groups that are geographically mixed (Fig. 2b, c), consistent with Tsang *et al.* [46]. Although Clade B is highly divergent and shares no haplotypes with Clade A at the mitochondrial level, the shallow population structure recovered reflects the geographic overlap of the haplotypes of the two clades, with the majority of the haplotypes being shared among the sampled areas (Fig. 2b). The pattern observed reflects a significant historical differentiation of the two clades and may represent an incipient speciation event. Lowered sea levels, as a consequence of historic glaciations, may have been responsible for the formation of allopatric lineages that have since come into secondary contact.

In the absence of a clear allopatric, geographic explanation that could have given rise to the two clades in *T. serrata*, it is important to consider that physiological differences could also result in genetic diversification. Field observations and physiological experiments on the mussel *Perna perna* indicated that the western lineage of the species is intolerant of high water temperatures, thus explaining its exclusion from the east coast [79]. Similarly, in the mudprawn *Upogebia africana*, larvae of the eastern lineage are unable to tolerate cooler waters and, thus, are unable to establish themselves in cool-water provinces [80]. The mtDNA data of *T. serrata* suggest that Clade B extends no further east than the border of the sub-tropical and temperate regions at Haga Haga, whereas Clade A can be found in the sub-tropical region (Fig. 1, Fig. 2b). This implies that Clade B may be constrained to more temperate environments, as a result of physiological intolerance. The annual mean sea surface temperatures experienced along the South African coastline ranges between 12°C on the west coast to 26°C on the east coast [81]. *Tetraclita serrata* displays regular and annual recruitment and Dye [82] revealed a high variation in spatial and inter-annual recruitment intensity along the east coast of South Africa. It was also found that recruitment density was related to mean abundance [82]. This suggests that larval dispersal and settlement may differ among regions. Along these lines, it is particularly notable that two distinct, species-level lineages of the Red Sea barnacle *Tetraclita rufotincta* occupy different vertical zones of the intertidal [83]. The genetic divergence in *T. serrata* may be explained by selective pressures due to ecological conditions, such as desiccation tolerance and predation. Given the geographic overlap of the two clades, it is possible that the genetic pattern found in *T. serrata* is the result of an ecological speciation process. Individuals belonging to Clades A and B may occupy different zones of the mid-intertidal (micro-habitat isolation), although this remains to be tested further.

Despite the clade structure present in the mtDNA data and a sequence divergence of ∼7.92%, the lack of geographic structure for Clades A and B and the overall lack of structure for the nuclear data suggest incipient speciation at best (Fig. 1, 2). This result is supported by Tsang *et al.* [46], who similarly found no genetic structure for nuclear DNA data (Histone 3) and no morphological differentiation. As the species is hermaphroditic, it can be ruled out that the lack of nuclear genetic structure is that of sex-biased dispersal, and is more than likely due to differences in the fixation rates of the two marker systems.

### Population genetic structure: associations with life-history characteristics

Several phylogeographic studies for southern African marine species suggest the presence of at least four marine phylogeographic breaks (see [19], [25]), of which we sampled across three (Cape Point, Cape Agulhas and the area between Port Elizabeth and the northern Transkei; see Fig. 1 of [19]). Although globally many studies have shown that life history characteristics such as pelagic larval duration (PLD) are not correlated with population genetic structure and gene flow [84], [85], planktotrophic larval dispersal of *T. serrata* may give some insight into the lack of structure recovered in this study. Even though the shared haplotypes found for the entire data set indicate that this brooding barnacle is able to disperse over long distances, suggestive of a long PLD, *T. serrata* does not form a panmictic population as several sampled localities are differentiated, even if Φ_ST_ values are low (Table 2). The presence of large numbers of haplotypes unique to certain regions (Table 1), particularly in the south-west coastal area (KO, WP, BB), provides evidence for population genetic structuring in the west and south-west coastal regions. The significant differentiation of Kommetjie (Cape Peninsula) and Betty's Bay (False Bay region) from other localities (Fig. 2b, Table 2) suggests that, at the finer geographic scale, dispersal may be restricted by water currents or by the local retention of larvae in the water column, as has been shown for other species [34], [86], [87], [88]. This is further supported by the signal of IBD for *T. serrata*.

### Gene flow in relation to ocean currents

The strength and directionality of gene flow in *T. serrata* show interesting correlations with oceanographic features along the South African coast. All west coast (except Port Nolloth) sampled localities experience gene flow primarily in the direction of the Benguela Current, while half of the localities sampled on the south-west coast (see below) experience gene flow in the direction of the Agulhas Current (Fig. 1). Ocean currents and prevailing wind direction have long been recognized as important factors affecting the distribution, abundance and genetic variation of marine benthic invertebrates with planktonic larvae [89], [90]. A pattern of unidirectional gene flow, which is much lower than relative gene flow estimates around Cape Point, may play a role in the genetic structuring of sampled localities on the south-west coast, evident in the haplotype network with haplotypes from the west coast and south-west coast sampling localities clustering together (Fig. 1). Such reduced gene flow was also shown for rocky shore fishes [22], [26] and an endemic sea urchin, *Parechinus angulosus* [24] strongly suggesting that gene flow is reduced for inshore coastal species in this region. Cape Point is also characterized as a region where a major biogeographic break occurs [19]. The barrier is thought to be the result of the distinct temperature regimes on either side of the peninsula, which may influence larval dispersal and/or survival in the different environments. By contrast, the signal is more mixed on the south and east coast, with bi-directional gene flow, suggesting that both the inshore, wind-driven countercurrents and the offshore Agulhas Current play a role in the distribution of larval *T. serrata* [19].

### Demographic history

The combination of high haplotype diversity and low nucleotide diversity is a typical signature of population expansion following a bottleneck [91], [92]. Fu's F_s_ simulations (Table 1) and the MK test, which was not significant, indicate that the observed deviations from equilibrium are not caused by natural selection and that *T. serrata* has undergone recent demographic change. The mtDNA haplotype network, showing several common haplotypes (Fig. 2), mismatch distributions (Table 1) and the timing of the most recent expansion event for *T. serrata* are indicative of a growing population. The population expansion of *T. serrata* predates the LGM, taking place 164 000 to 31 000 years ago, depending on the mutation rate and generation time used (Table 3). The BSP analyses agree with this (40 000 to 100 000 years ago, Fig. 4). Although many southern African marine taxa show patterns of post-LGM population growth [8], [20], [21], [93], [94], [95], [96], also confirmed by a number of barnacle species found elsewhere [5], [8], several studies among rocky shore marine species also show pre-LGM population expansions (Pacific Ocean: [8], [18], [97], [98], [99], [100]; Atlantic Ocean: [4]). This result suggests that the dramatic and frequent climatic changes of the Pleistocene, pre-dating the LGM, may have had a stronger impact in shaping the demographic and biogeographic history of *T. serrata* [8], [101]. Even if a more conservative approach was used, such as the mitochondrial mutation rate based on the divergence rate of 2% per million years [102], the demographic changes of *T. serrata* would still have predated the LGM. The two mutation rates that were used from barnacle genera were substantially greater than the 2% rate and still the population expansion of *T. serrata* predated the LGM. Therefore, despite the limitations of calculating the timing of expansion [27], the variation in mutation rates alone cannot account for the relatively deep demographic history for *T. serrata*.

Broadly, our results support Marko *et al.* [8] in that suspension feeders possessing planktonic larvae that occur in the mid-intertidal region show patterns of LGM persistence. The majority of studies to date on South African marine species showing patterns of post-LGM recolonization also possesses planktonic larval stages and is found subtidally, and is neither suspension feeders nor sessile species [7]. Nevertheless, these are certain traits worth considering for future research given that there are a number of barnacle species that possess planktonic larvae and live in rocky shore habitats showing clear population genetic signatures of regional persistence [4], [8], [18], [98].
